# Association between post-trauma platelet-lymphocyte ratio and nonunion in patients with extremity fractures: a multicenter retrospective cohort study

**DOI:** 10.1097/JS9.0000000000003265

**Published:** 2025-08-22

**Authors:** Chengsi Li, Zhenbang Yang, Puxin Yang, Zhiqiang Li, Tianrui Wang, Baorui Xing, Yubin Long, Yanbin Zhu, Yingze Zhang, Wei Chen

**Affiliations:** a Department of Orthopaedic Surgery, Hebei Medical University Third Hospital, Shijiazhuang, Hebei, People’s Republic of China; b Hand and Foot Surgery Department, Cangzhou People’s Hospital, Cangzhou, Hebei, People’s Republic of China; c Knee Preservation Center, Affiliated Hospital of Qingdao University, Qingdao, Shandong, People’s Republic of China; d Cangzhou Hospital of Integrated TCM-WM·Hebei, Cangzhou, Hebei, People’s Republic of China; e Department of Orthopedics 3, Baoding First Central Hospital, Baoding, Hebei, People’s Republic of China

**Keywords:** cohort study, extremity fractures, nonunion, platelet-lymphocyte ratio

## Abstract

**Background:**

Post-traumatic coagulation and inflammatory activation are associated with various clinical outcomes, and impairments of hematoma formation and inflammatory cell recruitment in the early phases potentially adversely affect bone healing. We aimed to explore and quantify the association between serum platelet-lymphocyte ratio (PLR) levels and postoperative nonunion in patients undergoing extremity fracture Surgery.

**Methods:**

This retrospective, multicenter cohort study analyzed data from five tertiary hospitals, including adult patients who underwent surgery for limb fractures between January 2020 and December 2022. The exposure was the post-trauma platelet-to-lymphocyte ratio (PLR), calculated by dividing the platelet count by the lymphocyte count. The outcome of interest was fracture nonunion, defined as the failure of fracture healing for a minimum of 9 months, without evidence of progression toward healing over the preceding 3 months, which was confirmed through a comprehensive review of medical records, telephone follow-ups, and outpatient assessments. Dose–response analysis using restricted cubic spline (RCS) function and multivariate logistic regression were conducted to evaluate the relationship between PLR and nonunion risk.

**Results:**

In total, 30 791 patients were included, with an average age of 49.1 ± 16.5 years, and 60.5% were males. Nonunion occurred in 656 patients (2.1%). RCS analysis showed a significant negative non-linear dose–response relationship between PLR and nonunion risk (*P*-nonlinearity < 0.001, *P*-overall < 0.001), and the cutoff value for the risk-protective effect was 145.0. Compared to high PLR levels (≥145.0), lower PLR levels (<145.0) were associated with higher nonunion risk (odds ratio = 1.613; 95% CI, 1.379–1.887; *P* < 0.001); and this risk effect remained evident after adjustment (adjusted odds ratio = 1.265; 95% CI, 1.057–1.515; *P* = 0.010). Results are robust across several prespecified sensitivity or exploratory analyses. Subgroup analyses revealed heterogeneity of association according to surgery site and fixation methods (*P* values for interaction < 0.050).

**Conclusion:**

This study demonstrates that reduced post-trauma PLR levels are associated with an increased risk of nonunion, serving as a potential biomarker for risk stratification and personalized intervention planning during the critical bone healing window.

## Introduction

Despite significant advancements in minimally invasive techniques, internal fixation materials and technologies, and perioperative comprehensive care, nonunion remains one of the most challenging and debilitating complications following fracture surgery^[1]^. According to international literature, fracture nonunion occurs in 1.9% to 10% of surgically treated fracture cases^[2–4]^, causing persistent pain in 72% of patients and work incapacity in 56% within a year^[5]^. Currently, a consensus has been established on the management of nonunion, however, targeted and effective tools or metrics for the early prediction of nonunion are lacking. Existing methods, including imaging, clinical scoring systems, and serum biomarkers, have clear limitations. Imaging techniques are primarily used for diagnostic follow-up and lack predictive utility. Furthermore, repeat imaging poses challenges for patients with limited mobility due to fractures^[6–8]^. Although several clinical scoring systems have been proposed, they are often overly complex and lack external validity, limiting their clinical applicability. While numerous serum biomarkers associated with bone healing have been identified in preclinical studies, their clinical relevance and applicability remain uncertain^[9,10]^.

During the early post-fracture phase, platelets play a critical role by adhering, activating, and aggregating to form stable hematomas within the fracture space and releasing pro-inflammatory mediators, thereby initiating the inflammatory cascade^[11]^. Subsequently, lymphocytes infiltrate the hematoma, modulating the temporal dynamics of the inflammatory phase through cytokine secretion, which, in turn, directly impacts the subsequent stages of bone healing^[10]^. Theoretically, post-traumatic platelets and lymphocytes may significantly influence the healing process and serve as potential biomarkers for predicting the risk of nonunion. As a composite biomarker reflecting both platelet and lymphocyte counts, the platelet-lymphocyte ratio (PLR) has shown substantial prognostic value in predicting clinical outcomes, including early neurological deterioration, heart failure and death across a range of acute injury scenarios^[12–16]^. In the domain of orthopedics, PLR has also demonstrated considerable promise as a predictive marker for outcomes following major fracture surgeries, as well as hip and knee arthroplasty^[17–19]^. Unlike traditional diagnostic modalities such as imaging or clinical scoring systems, PLR provides an objective, observer-independent metric that can be assessed early post-fracture. Given the global incidence of over 178 million new fractures annually^[20]^, the relatively high frequency of developing nonunion^[3,4]^, and the lack of reliable early predictive methods, it becomes imperative to investigate the relationship between PLR and nonunion, as well as to evaluate its potential utility in forecasting this complication.

Therefore, our study aims to explore and quantify the association between serum PLR levels and postoperative nonunion in fracture patients. This cohort study has been reported in line with the STROCSS guidelines^[21]^.

## Methods

### Study design and data collection

This was a retrospective, multicenter cohort study conducted across five tertiary hospitals, comprising two university hospitals and three teaching hospitals, four Level I and one Level II trauma center, serving 28.4 million inhabitants. The study was conducted from January 2020 to December 2022. Data were collected from each institution’s electronic medical records by well-trained researchers who were independent of patient care. The ethics committees of each institution approved the study and waived the need for informed consent because of the retrospective nature and deidentification of patient data. The study adhered to the Declaration of Helsinki^[22]^.

### Population

The study included 48 301 patients who: (1) were aged 18 years or older; (2) had index extremity fractures (identified by *International Classification of Diseases, 9th Revision, Clinical Modification* [ICD-9-CM] codes 810.00-829.10^[23]^, and confirmed by radiographs); (3) underwent surgical procedures; (4) had complete platelet and lymphocyte count data; and (5) completed a follow-up assessment of at least 12 months after surgery.

The exclusion criteria were: (1) periprosthetic, stress, or pathologic fractures; (2) fractures of the same bone within 12 months before the index fractures; (3) unavailability of laboratory test data of interest within 48 hours following trauma; (4) preoperative infection; (5) use of antiplatelet therapy within 2 weeks before admission; (6) perioperative blood transfusions; (7) receipt of immunosuppressive therapy, chemotherapy, or radiotherapy within the past 12 months; (8) new fractures sustained during the 12-month follow-up period; or (9) incomplete 12-month follow-up information.

**HIGHLIGHTS**

- This multicenter study of 30 791 patients identified a platelet-lymphocyte ratio (PLR) <145.0 within 48 hours post-injury as a significant independent predictor of fracture nonunion risk, increasing the likelihood by 26.5%.
- The findings validate the clinical relevance of post-traumatic coagulation and inflammatory responses, establishing PLR as the first widely accessible biomarker for predicting bone healing outcomes.
- Early PLR assessment enables clinicians to stratify patient risk effectively, facilitating targeted interventions and potentially reducing the need for costly imaging and repeat surgical procedures.

### Measurement of PLR

Fasting blood samples were collected from peripheral veins typically on the first morning after admission. In cases warranting emergency surgeries, blood samples were obtained during the initial clinical assessment. According to the manufacturers’ standard operating procedures, two university-affiliated hospitals used the DxH 800 Hematology Analyzer (Beckman Coulter, USA), while the other three teaching hospitals employed the DxH 560 Hematology Analyzer (Beckman Coulter, USA). Platelet and lymphocyte counts were obtained through complete blood count (CBC) analysis of peripheral blood samples using these instruments. The PLR was calculated by dividing the absolute platelet count (×10^9^ cells/L) by the absolute lymphocyte count (×10^9^ cells/L).

### Outcomes

The outcome was nonunion, defined by the U.S. Food and Drug Administration (FDA) as a fracture that persists for a minimum of 9 months without signs of healing for three months^[1]^. Each reported nonunion was identified using the ICD-9-CM code 733.82 and confirmed by reviewing medical records, telephone follow-ups, and outpatient follow-up assessments. Suspicious cases were adjudicated by an expert review committee consisting of two orthopedic surgeons and one imaging specialist.

### Covariates

Patient-level confounders known to predict nonunion or affect bone healing were identified a priori, including demographic characteristics (age, sex, body mass index [BMI], place of residence and history of surgery); lifestyle factors (smoking status and alcohol consumption); and comorbidities (hypertension, diabetes, cardio-cerebrovascular diseases, pulmonary, kidney and liver diseases)^[3]^. Laboratory parameters, including red blood cell (RBC) count, white blood cell (WBC) count, albumin (ALB)^[24,25]^, creatinine (CREA), and activated partial thromboplastin time (APTT)^[11]^, reflecting inflammatory and systemic conditions that may affect PLR levels, were included. Clinical characteristics empirically associated with bone healing, such as injury type, surgery site, emergency surgery, wound classification, surgical delay, American Society of Anesthesiologists (ASA) class, anesthesia type, surgery type, duration of surgery, blood transfusion type, surgical antibiotic prophylaxis (SAP) duration, and year of surgery, were also considered.

### Statistical analysis

Continuous variables were presented as mean ± standard deviation or median [Q1–Q3], based on the normal or skewed distribution, and compared using student’s t-test or Mann–Whitney U test, as appropriate. Categorical variables were expressed as numbers (%) and analyzed with the chi-square or Fisher’s exact test. Missing data were handled by listwise deletion. Before any regression analyses and modeling, multicollinearity was tested using the multiple linear regression, and variables with a variance inflation factor (VIF) ≥3 were excluded from further analyses^[26]^.

The restricted cubic splines (RCS) curve was to assess the dose-effect relationship between PLR levels. In RCS, four-knots was used, and threshold for the risk-protective effect of PLR level on nonunion was determined by considering the intersection point between odds ratio (OR = 1.00) and the x-axis, and patients were subsequently dichotomized into high and low PLR groups.

Multivariable binary logistic regression was used to calculate the odds ratio and its 95% confidence interval (CI) of nonunion associated with high PLR compared to low PLR level, using the *Enter method* and including variables with a univariable *P* value <0.20. The goodness of fit was assessed using the *Hosmer–Lemeshow* test with *P* value >0.05 indicating an acceptable result.

To test the robustness of the main results several sensitivity and exploratory analyses were conducted by excluding patients: (1) with PLR levels in the extreme 2.5% at both ends of the distribution; (2) with delayed surgery (more than 2 weeks from injury to surgery); (3) developing post-operative surgical site infections; and (4) having received temporary external fixation before definitive internal fixation; and by (5) adding ALP alkaline phosphatase level for adjustment; (6) treating surgeon experience as a fixed effect; and (7) using a composite of fracture nonunion or delayed union as dependent outcome.

Heterogeneity of the associations was assessed by adding an interaction term between PLR level and the following prespecified subgroups: sex (man vs. woman), age (<65 vs. ≥65 years), BMI (<28 vs ≥28 kg/m^2^), surgery site (upper limb, vs. lower limb, vs. multiple), injury type (open vs. closed fractures) and fixation methods (plate, vs. screw/wire, vs. intramedullary nail, vs external fixator).

All analyses were performed using R software (R Foundation for Statistical Computing version 4.3.2), and a two-tailed *P* <0.05 indicates a statistically significant difference.

## Results

**Figure 1.**
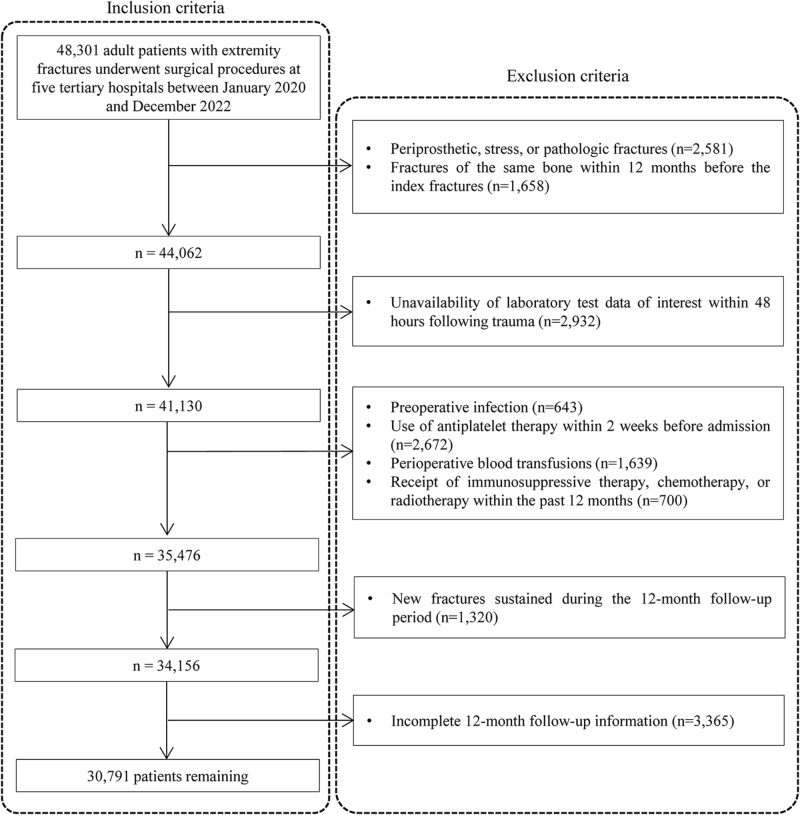
Patient inclusion and exclusion flow chart.

During the study period, 48 301 patients with extremity fractures presented to a participating hospital and 17 510 were excluded (Fig. 1). A total of 30 791 patients were included in the analysis, 60.5% were male, and the average age was 49.1 ± 16.5 years. A total of 656 patients were diagnosed with nonunion, indicating an accumulated rate of 2.1% (95% CI, 2.0–2.3). The median platelet counts, lymphocytes counts and PLR were 219.2 (179.1–268.0) × 10^9^ /L, 1.51 (1.12–1.96) × 10^9^ cells/L, and 144.1 (107.7–201.8), respectively. And the median time from injury to sampling was 1.0 (0.0–1.0) days.

**Figure 2.**
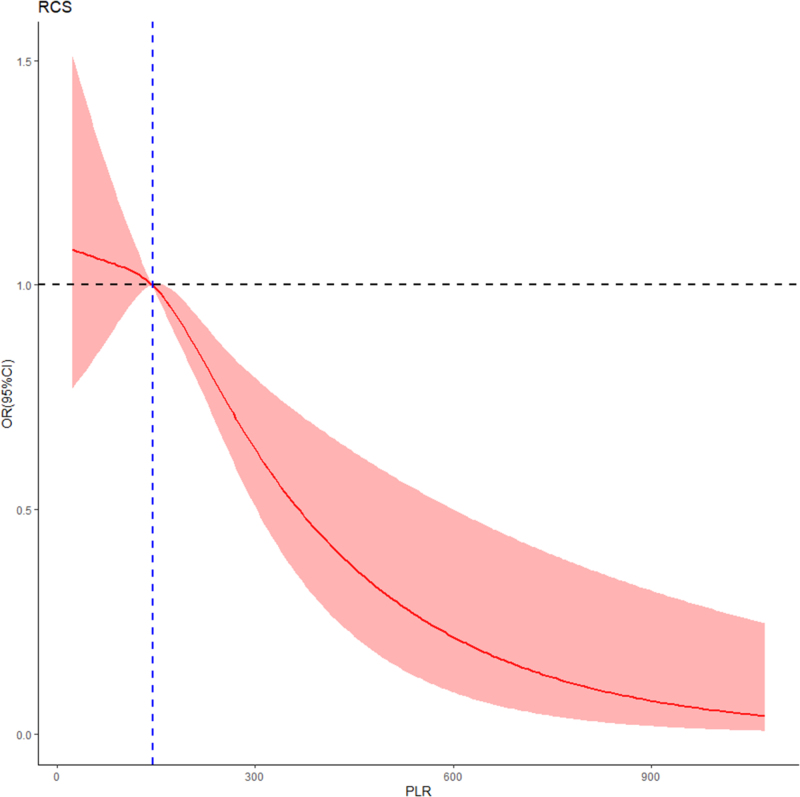
Dose-effect relationship between post-trauma platelet-lymphocyte ratio (PLR) and the incidence of nonunion. Evaluated using a restricted cubic spline model with four knots and adjusted by age, sex, body mass index (BMI), place of residence and history of surgery, smoking status, alcohol consumption, hypertension, diabetes, cardio-cerebrovascular diseases, pulmonary, kidney and liver diseases, red blood cell (RBC) count, white blood cell (WBC) count, albumin (ALB), creatinine (CREA), activated partial thromboplastin time (APTT), injury type, surgery site, emergency surgery, wound classification, surgical delay, American Society of Anesthesiologists (ASA) class, anesthesia type, surgery type, duration of surgery, blood transfusion type, surgical antibiotic prophylaxis (SAP) duration, year of surgery, and the PLR. The red line and the area between the light-red shade represents the estimated values and their corresponding 95% CI, respectively. The cutoff point of the risk protection effect is 145.0. CI, confidence interval.

In RCS, there was a significant negative non-linear dose–response relationship between post-trauma PLR levels and the risk of nonunion (*P*-nonlinearity < 0.001, *P*-overall < 0.001), and the cutoff value for the risk-protective effect of PLR on nonunion was 145.0 (Fig. 2). Significant differences were observed between the high PLR (>145.0) and low PLR (<145.0) groups at baseline in terms of age, sex, BMI, place of residence, smoking status, hypertension, diabetes, cardio-cerebrovascular diseases, pulmonary, kidney and liver diseases, RBC, WBC, ALB, CREA, APTT, injury type, surgery site, wound class, surgical delay, ASA class, anesthesia type, surgery type, duration of surgery, and blood transfusion type (*P* < 0.050; Table 1).

**Figure 3.**
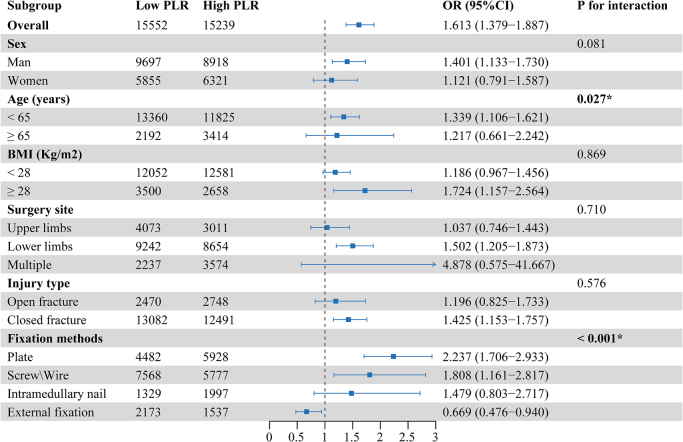
The association of post-trauma platelet-lymphocyte ratio levels with the risk of nonunion in various subgroups.

**Table 1:** Baseline characteristics of participants^a^

Characteristic	Low PLR	High PLR	*P*
**Total**	15 552	15 239	
**PLR**	108.06 (89.40–125.42)	202.69 (168.67–264.25)	<0.001
**Nonunion**			<0.001
No	15 150 (97.4%)	14 985 (98.3%)	
Yes	402 (2.6%)	254 (1.7%)	
**Demographic**			
Age (years)			<0.001
18–44	6849 (44.04%)	5560 (36.49%)	
45–64	6511 (41.87%)	6265 (41.11%)	
65–79	1726 (11.10%)	2444 (16.04%)	
≥80	466 (3.00%)	970 (6.37%)	
Sex			<0.001
Man	9697 (62.35%)	8918 (58.52%)	
Women	5855 (37.65%)	6321 (41.48%)	
BMI^b^ (kg/m^2^)			<0.001
<18.5	327 (2.10%)	479 (3.14%)	
18.5–23.9	5227 (33.61%)	5862 (38.47%)	
24–27.9	6498 (41.78%)	6240 (40.95%)	
≥28	3500 (22.51%)	2658 (17.44%)	
Place of residence			<0.001
Country	9649 (62.04%)	10 182 (66.82%)	
Urban	5903 (37.96%)	5057 (33.18%)	
History of surgery			0.909
No	11 846 (76.17%)	11 616 (76.23%)	
Yes	3706 (23.83%)	3623 (23.77%)	
**Lifestyle factors**			
Smoking status			<0.001
Never smoker	11 706 (75.27%)	11 541 (75.73%)	
Former smoker	3234 (20.79%)	2979 (19.55%)	
Current smoker	612 (3.94%)	719 (4.72%)	
Alcohol consumption			0.250
No	13 115 (84.33%)	12 778 (83.85%)	
Yes	2437 (15.67%)	2461 (16.15%)	
**Comorbidities**			
Hypertension			<0.001
No	12 607 (81.06%)	11 665 (76.55%)	
Yes	2945 (18.94%)	3574 (23.45%)	
DM			<0.001
No	13 808 (88.79%)	12 939 (84.91%)	
Yes	1744 (11.21%)	2300 (15.09%)	
CBVD			<0.001
No	14 568 (93.67%)	13 484 (88.48%)	
Yes	984 (6.33%)	1755 (11.52%)	
CVD			<0.001
No	13 978 (89.88%)	13 213 (86.71%)	
Yes	1574 (10.12%)	2026 (13.29%)	
COPD			<0.001
No	15 425 (99.18%)	15 025 (98.60%)	
Yes	127 (0.82%)	214 (1.40%)	
CKD			<0.001
No	15 376 (98.87%)	14 967 (98.22%)	
Yes	176 (1.13%)	272 (1.78%)	
CLD			<0.001
No	14 875 (95.65%)	14 713 (96.55%)	
Yes	677 (4.35%)	526 (3.45%)	
**Laboratory examination**			
RBC^c^	4.37 (3.94–4.76)	3.91 (3.43–4.35)	<0.001
Normal value	12 408 (79.78%)	8793 (57.70%)	<0.001
Decreased value	2778 (17.86%)	6325 (41.51%)	
Elevated value	366 (2.35%)	121 (0.79%)	
WBC	7.42 (5.98–9.39)	8.37 (6.52–10.57)	<0.001
<10^a^10^9^/L	12 541 (80.64%)	10 539 (69.16%)	<0.001
≥10^a^10^9^/L	3011 (19.36%)	4700 (30.84%)	
ALB	41.78 (38.20–44.80)	38.10 (33.20–42.30)	<0.001
<35 g/L	13 439 (86.41%)	10 184 (66.83%)	<0.001
≥35 g/L	2113 (13.59%)	5055 (33.17%)	
CREA^d^	63.40 (53.94–73.00)	58.63 (50.00–68.50)	<0.001
Normal value	15 512 (99.74%)	15 154 (99.44%)	<0.001
Abnormal value	40 (0.26%)	85 (0.56%)	
APTT (s)	30.20 (28.10–32.60)	29.60 (27.50–32.00)	<0.001
**Treatment**			
Injury type^e^			<0.001
Open fracture	2470 (15.88%)	2748 (18.03%)	
Closed fracture	13 082 (84.12%)	12 491 (81.97%)	
Surgery site			<0.001
Upper limb	4073 (26.19%)	3011 (19.76%)	
Lower limb	9242 (59.43%)	8654 (56.79%)	
Multiple	2237 (14.38%)	3574 (23.45%)	
Emergency surgery			0.593
Non-emergent	14 196 (91.28%)	13 884 (91.11%)	
Emergent	1356 (8.72%)	1355 (8.89%)	
Wound class			0.010
Clean	13 523 (86.95%)	13 357 (87.65%)	
Clean-contaminated	1585 (10.19%)	1408 (9.24%)	
Contaminated & dirty	444 (2.85%)	474 (3.11%)	
Surgical delay (day)	4.00 (3.00–6.00)	5.00 (3.00–9.00)	<0.001
ASA class			<0.001
I	2716 (17.46%)	2097 (13.76%)	
II	10 446 (67.17%)	9934 (65.19%)	
III	2249 (14.46%)	3040 (19.95%)	
IV&V	141 (0.91%)	168 (1.10%)	
Anesthesia type			<0.001
General	10 365 (66.65%)	8605 (56.47%)	
Others	5187 (33.35%)	6634 (43.53%)	
Surgery type^f^			<0.001
Others^g^	641 (4.12%)	1276 (8.37%)	
Implant	8666 (55.72%)	11 203 (73.52%)	
Replacement	6245 (40.16%)	2760 (18.11%)	
Duration of surgery			<0.001
≤60 min	1947 (12.52%)	889 (5.83%)	
61–119 min	6892 (44.32%)	5430 (35.63%)	
120–179 min	4272 (27.47%)	4977 (32.66%)	
≥180 min	2441 (15.70%)	3943 (25.87%)	
Blood transfusion type			<0.001
None	14 234 (91.53%)	12 432 (81.58%)	
Autologous & allogeneic	112 (0.72%)	240 (1.57%)	
Allogeneic	1076 (6.92%)	2304 (15.12%)	
Autologous	130 (0.84%)	263 (1.73%)	
SPA duration (day)	2.00 (1.00–4.00)	2.00 (1.00–4.00)	0.100
Year of surgery	15 552	15 239	0.199
2020	5150	5132	
2021	5211	5099	
2022	5191	5008	

a Values are n (%) for categorical variables, and median [Q1–Q3] for continuous variables. χ2 test or Fisher exact test was used for categorical variables, and Mann–Whitney test was used for continuous variables.

BMI = body mass index; DM = Diabetes mellitus; CBVD = cerebrovascular disease; CVD = cardiovascular disease; COPD = chronic obstructive pulmonary disease; CKD = chronic kidney disease; CLD = chronic liver disease; RBC = red blood cell; WBC = white blood cell; ALB = albumin; CREA = creatinine; APTT = activated partial thromboplastin time; ASA = American Society of Anesthesiologists; SAP = surgical antibiotic prophylaxis.

^b^ BMI was used to divide participants into underweight (BMI, <18.5), normal weight (BMI, 18.5–23.9), overweight (BMI, 24.0–27.9), and obese (BMI, ≥28.0) groups according to the Working Group on Obesity in China (WGOC) cutoffs.

^c^ RBC were classified as normal, decreased, or elevated based on the following ranges: 4.5–5.5 × 10^12^/L for males and 3.5–5.0 × 10^12^/L for females.

^d^ CREA were classified as normal or abnormal based on the following ranges: 59–104 μmol/L for males and 45–84 μmol/L for females.

^e^ Injury type: classifying based on the admission diagnosis, include closed and open fracture.

^f^ Surgery type: classifying based on the common orthopedic surgeries’ characteristics.

^g^ Includes debridement, osteotomy, arthroscopy, bone grafting.

No multicollinearity was detected among the covariates (Supplementary Digital Content, Table S1, available at: http://links.lww.com/JS9/E934). Compared to high PLR level (≥145.0), low PLR level (<145.0) was significant associated with increased nonunion risk (odd ratio = 1.613; 95% CI, 1.379-1.887; *P* < 0.001); and this risk effect remained evident in covariate adjusted multivariate analysis (adjusted odd ratio [aOR] = 1.265; 95% CI, 1.057–1.515; *P* = 0.010; Table 2).

**Table 2:** Covariate adjusted multivariate logistic regression of PLR and other covariates for nonunion

Characteristic	B	Wald	*P*	OR	95% CI	
					Lower	Upper
PLR group < 145.0	0.235	6.551	0.010	1.265	1.057	1.515
Sex (woman)	−0.510	18.089	0.000	0.600	0.475	0.760
Place of residence (urban)	−0.634	43.307	0.000	0.531	0.439	0.641
Former smoker	−0.097	0.563	0.453	0.907	0.704	1.170
Current smoker	0.430	6.186	0.013	1.537	1.095	2.157
Alcohol consumption	0.159	1.406	0.236	1.173	0.901	1.525
Hypertension	−0.186	2.443	0.118	0.830	0.657	1.048
DM	−0.052	0.136	0.712	0.949	0.719	1.253
CBVD	−0.537	6.530	0.011	0.585	0.387	0.882
CKD	−1.040	2.047	0.153	0.353	0.085	1.470
CLD	−0.296	1.440	0.230	0.744	0.459	1.206
CREA	−0.002	0.513	0.474	0.998	0.991	1.004
ALB	−0.035	14.034	0.000	0.966	0.949	0.984
RBC	0.812	93.822	0.000	2.253	1.912	2.656
History of surgery	1.277	237.621	0.000	3.584	3.047	4.216
Emergency surgery	−2.138	58.029	0.000	0.118	0.068	0.204
Injury type (closed fracture)	−0.461	15.170	0.000	0.631	0.500	0.795
Surgery site (lower limb)	0.609	21.617	0.000	1.839	1.422	2.377
Surgery site (multiple)	−1.982	31.387	0.000	0.138	0.069	0.276
Surgery type (ORIF surgery)	0.576	6.772	0.009	1.779	1.153	2.745
Surgery type (others)	0.461	3.952	0.047	1.586	1.006	2.499
Anesthesia type (local)	−0.095	0.402	0.526	0.909	0.678	1.220
Anesthesia type (others)	−0.420	7.894	0.005	0.657	0.491	0.881
ASA class II	−0.211	3.728	0.054	0.810	0.653	1.003
ASA class III	−0.365	5.302	0.021	0.694	0.509	0.947
ASA class IV&V	−1.305	3.158	0.076	0.271	0.064	1.144
Wound class (clean-contaminated)	0.410	9.238	0.002	1.507	1.157	1.963
Wound class (contaminated & Dirty)	0.807	12.758	0.000	2.242	1.439	3.491
Duration of surgery	0.001	11.827	0.001	1.001	1.000	1.001
Blood transfusion type (autologous & allogeneic)	1.446	21.092	0.000	4.246	2.291	7.869
Blood transfusion type (allogeneic)	0.821	33.164	0.000	2.274	1.719	3.007
Blood transfusion type (autologous)	1.600	39.437	0.000	4.955	3.007	8.166
Year of surgery (2021)	0.030	0.086	0.769	1.030	.846	1.254
Year of surgery (2022)	0.130	1.726	0.189	1.139	.938	1.383
Constant	−6.010	156.721	0.000	0.002		

Abbreviations: PLR = platelet-to-lymphocyte ratio; OR = odds ratio; CI = confidence interval; DM = diabetes mellitus; CBVD = cerebrovascular disease; CKD = chronic kidney disease; CLD = chronic liver disease; CREA = creatinine; ALB = albumin; RBC = red blood cell count; ORIF = open reduction and internal fixation; ASA = American Society of Anesthesiologists classification.

Findings from sensitivity and exploratory analyses were broadly consistent with the main analyses (Supplementary Digital Content, Table S2, available at: http://links.lww.com/JS9/E934; Supplementary Digital Content, Table S3, available at: http://links.lww.com/JS9/E934). Subgroup analyses revealed statistically significant interactions between low PLR levels and age (with an aOR of 1.339 [95% CI, 1.106–1.621] in patients aged <65; *P* for interaction = 0.027; Fig. 3) as well as fixation methods (aOR 2.237 [95% CI, 1.706–2.933] for plate fixation, aOR 1.808 [95% CI, 1.161–2.817] for screw/wire fixation, and aOR 0.669 [95% CI, 0.476–0.940] for external fixation, *P* for interaction < 0.001).

## Discussion

In this study, we found a negative dose-effect relationship between serum PLR levels and nonunion, with a risk-protective threshold established at 145.0. After adjusting for confounders, low-level PLR was independently associated with a 26.5% increase in the risk of nonunion. This finding remained robust in sensitivity and exploratory analyses. Subgroup analyses revealed significant heterogeneity based on age and fixation methods.

The overall nonunion rate in our cohort was 2.1%, notably lower than the 5% to 10% range typically reported in the literature on fracture nonunion^[1,3]^. Several factors may account for this discrepancy. First, our study implemented more stringent exclusion criteria, systematically excluding patients with established high-risk factors for nonunion, such as preoperative infections, immunosuppressive therapies (including chemotherapy and radiotherapy), or fractures of non-traumatic origins (e.g., stress, pathological, or periprosthetic fractures). Second, our analysis was confined to long bone fractures of the extremities, which generally exhibit a lower nonunion risk compared to fractures in high-risk anatomical sites such as the scaphoid or talus^[3,27]^. Lastly, we included only those patients whose index fracture and subsequent nonunion were both managed within the same participating hospital, thereby ensuring more standardized follow-up and minimizing potential reporting biases.

The formation of post-trauma hematoma and activation of the inflammatory response are well-recognized initiators of fracture healing^[11]^. Our finding that lower post-trauma PLR levels are associated with an increased risk of nonunion can be explained by platelet-mediated phase-specific healing dysfunction and lymphocyte-driven microenvironmental imbalance^[1]^. First, platelets release key growth factors such as PDGF, TGF-β, and VEGF, which orchestrate mesenchymal stem cell recruitment, endothelial activation, and the transition from hematoma to callus formation. Inadequate platelet availability compromises this early reparative niche, impairs vascular invasion, and delays osteoblast-driven mineralization^[28]^. Additionally, a reduction in platelet disrupts the GPIb-CD11b axis, leading to a prolonged pro-inflammatory state dominated by M1 macrophages, which in turn impair osteogenic differentiation and exacerbate tissue degradation at the fracture site^[29]^. Moreover, lymphocytes play a critical role in resolving inflammation and coordinating bone repair by directly interacting with osteoblasts and osteoclasts^[30]^. A reduced PLR, especially when attributable to an expansion of pro-inflammatory lymphocyte subsets, may impair the phased transition from pro-inflammatory to pro-regenerative responses and compromise crosstalk between osteogenesis and angiogenesis, thereby increasing the risk of nonunion^[31]^.

Previous studies have examined the ability of PLR to predict clinical outcomes following acute injury or major surgery^[12–19]^. For example, Chen *et al*^[14]^ prospectively evaluated 448 patients with first-ever acute ischemic stroke and found that a high admission PLR was independently associated with an adverse functional outcome at 3 months, with an optimal cutoff value of 141.5 (*P*-trend < 0.001). Similarly, Wang *et al*^[19]^ retrospectively analyzed 460 hip fracture patients and reported that a high admission PLR (≥189) was associated with an increased one-year all-cause mortality rate after a median follow-up of 32.0 months (range, 12.0–75.4 months). These studies highlight the broad predictive utility of PLR for prognosis across various types of acute injury. Interestingly, the predictive trend of PLR for nonunion contrasts sharply with its association with most other healing outcomes, a divergence that may reflect the functional heterogeneity of pathophysiological processes captured by this biomarker across different tissues. In conditions such as acute aortic dissection, ischemic stroke, and renal injury, elevated PLR predominantly serves as an indicator of acute systemic inflammation and stress^[12,14,15]^. Here, high PLR levels are associated with microvascular dysfunction, exacerbated secondary tissue injury, and poor prognosis, aligning with disease progression. In contrast, during bone healing, early hematoma formation marks the initiation of repair, establishing a localized microenvironment enriched with platelets, coagulation factors, and cytokine. This milieu orchestrates the recruitment of inflammatory cells, paving the way for chondrogenesis and subsequent ossification^[11]^. As existing biomarkers for early prediction of bone healing remain largely confined to preclinical studies^[9,10]^, this study expands the scope of early prediction of bone nonunion in real-world clinical settings.

Sensitivity and exploratory analyses yielded results consistent with the primary findings, reinforcing the robustness of our conclusions and suggesting that the predictive value of post-trauma PLR for nonunion risk is independent of subsequent interventions and unlikely to be affected by extreme values or adverse events such as infection. Subgroup analyses demonstrated significant heterogeneity across age groups and fixation methods, suggesting that patients within these specific subgroups may be at a heightened risk of nonunion and could benefit from targeted risk stratification and proactive intervention strategies. However, given that adjustments for multiple comparisons were not applied, these findings should be interpreted with caution, particularly in subgroups with potential confounding effects that may obscure the main associations, such as those receiving external fixation^[32]^. Further validation in future studies is warranted.

### Research implications

Our findings hold significant clinical implications with potential to refine both prognostic strategies and therapeutic approaches. First, platelet and lymphocyte counts are routinely measured laboratory parameters that are readily accessible, objective, and inexpensive, with no additional cost burden to patients. Second, PLR has the potential to function as a robust, independent prognostic biomarker, and also, its integration into clinical prediction models could substantially enhance risk stratification, offering a valuable tool to improve the predictive accuracy for fracture nonunion. Third, assessing PLR levels within the first 48 hours post-trauma provides a critical window for early, targeted intervention. For patients with PLR<145.0 (reflecting platelet insufficiency or dysregulated inflammation), early adjunctive platelet supplementation (e.g., PRP) combined with stable fixation may synergistically modulate the inflammatory-reparative cascade. Evidence showed PRP can accelerate osteogenesis in 67% of preclinical models and reduce healing time by 3–4 weeks in select clinical scenarios (e.g., long bone nonunions), particularly for high-risk cohorts (diabetics, elderly)^[33,34]^. In addition, extracorporeal shock wave therapy, pulsed electromagnetic fields, and acupuncture may be considered as potential treatment options^[35]^, but there is currently no conclusive or consistent evidence supporting their clinical effectiveness.

Our study has several limitations. First, its retrospective design may have affected the accuracy and completeness of data collection, potentially introducing recall bias, selection bias, or unmeasured confounders. Although rigorous data extraction protocols and statistical adjustments were implemented, the possibility of missing or inconsistently recorded variables cannot be entirely ruled out. Second, PLR was assessed solely based on admission values, without accounting for subsequent measurements or temporal fluctuations, particularly post-surgical changes. Additionally, the exact time of trauma, down to the hour, was not consistently available in medical records, making it challenging to determine the precise temporal trajectory of PLR dynamics following trauma. Instead, we applied a pragmatic 48-hour threshold as a surrogate marker. Future studies examining the peak and longitudinal trends of PLR fluctuations may offer more clinically relevant insights. Third, the five participating centers used two different generations of hematology analyzers from the same manufacturer to obtain parameters for PLR calculation. Although all centers adhered to strict internal quality control procedures, we also compared key hematological parameters (PLT, LYM, and PLR) across centers by age and gender groups, and found no substantial differences, supporting overall measurement consistency. Nevertheless, previous studies have suggested that inter-instrument variability may exist among different hematology analyzers^[36]^. Therefore, future studies are warranted to validate our findings under standardized laboratory conditions. Fourth, although this study was conducted across multiple centers, all participating institutions were tertiary referral hospitals, with four designated as Level I trauma centers. As a result, the findings may not fully reflect the broader spectrum of fracture severity or standard surgical management across diverse healthcare settings, potentially limiting generalizability to other patient populations or institutions.

## Conclusion

This study demonstrates that reduced PLR levels in the early post-traumatic phase independently predict nonunion risk in extremity fracture patients, serving as a potential biomarker for risk stratification and personalized intervention planning during the critical bone healing window. Further prospective studies with serial PLR monitoring are warranted to validate its clinical applicability.

## Data Availability

The datasets generated and/or analyzed during the current study are not publicly available due to their containing information that could compromise the privacy of research participants but are available from the corresponding author on reasonable request

## Research registration unique identifying number (UIN)

Research registry UIN: researchregistry11155 (https://researchregistry.knack.com/researchregistry#home/registrationdetails/67f9fb651f714f02bfd13407/).
